# The plant coumarins auraptene and lacinartin as potential multifunctional therapeutic agents for treating periodontal disease

**DOI:** 10.1186/1472-6882-12-80

**Published:** 2012-06-28

**Authors:** Annie Marquis, Salvatore Genovese, Francesco Epifano, Daniel Grenier

**Affiliations:** 1 Groupe de Recherche en Écologie Buccale, Faculté de Médecine Dentaire, Université Laval, 2420 Rue de la Terrasse, Quebec City,, QC, Canada, G1V 0A6; 2 Dipartimento di Science del Farmaco, Università G. D'Annunzio, Via Dei Vestini 31, 66013, Chieti Scalo, Chieti,, Italy

**Keywords:** Coumarins, Auraptene, Lacinartin, Antibacterial, Anti-adherence, Anti-inflammatory

## Abstract

**Background:**

Periodontal diseases are bacterial infections leading to chronic inflammation disorders that are frequently observed in adults. In the present study, we evaluated the effect of auraptene and lacinartin, two natural oxyprenylated coumarins, on the growth, adherence properties, and collagenase activity of *Porphyromonas gingivalis*. We also investigated the capacity of these compounds to reduce cytokine and matrix metalloproteinase (MMP) secretion by lipopolysaccharide (LPS)-stimulated macrophages and to inhibit MMP-9 activity.

**Methods:**

Microplate dilution assays were performed to determine the effect of auraptene and lacinartin on *P. gingivalis* growth as well as biofilm formation stained with crystal violet. Adhesion of FITC-labeled *P. gingivalis* to oral epithelial cells was monitored by fluorometry. The effects of auraptene and lacinartin on LPS-induced cytokine and MMP secretion by macrophages were determined by immunological assays. Fluorogenic assays were used to evaluate the capacity of the two coumarins to inhibit the activity of *P. gingivalis* collagenase and MMP-9.

**Results:**

Only lacinartin completely inhibited *P. gingivalis* growth in a complex culture medium. However, under iron-limiting conditions, auraptene and lacinartin both inhibited the growth of *P. gingivalis*. Lacinartin also inhibited biofilm formation by *P. gingivalis* and promoted biofilm desorption. Both compounds prevented the adherence of *P. gingivalis* to oral epithelial cells, dose-dependently reduced the secretion of cytokines (IL-8 and TNF-α) and MMP-8 and MMP-9 by LPS-stimulated macrophages, and inhibited MMP-9 activity. Lacinartin also inhibited *P. gingivalis* collagenase activity.

**Conclusions:**

By acting on multiple targets, including pathogenic bacteria, tissue-destructive enzymes, and the host inflammatory response, auraptene and lacinartin may be promising natural compounds for preventing and treating periodontal diseases.

## Introduction

Periodontal diseases are chronic inflammatory disorders of bacterial origin that affect tooth-supporting tissues [1]. It is estimated that 5% to 20% of any population suffers from severe, generalized periodontitis, while mild to moderate periodontitis affects a majority of adults [2]. These diseases are mixed infections induced by a specific group of Gram-negative anaerobic bacteria called periodontopathogens [3]. Of the over 700 bacterial species that have been identified in the oral cavity [4] only a few are associated with periodontitis, including *Porphyromonas gingivalis*[5]. This bacterial species produces a number of virulence factors that contribute to host colonization, immune defense system neutralization, and periodontal tissue destruction [5]. High numbers of *P. gingivalis*, together with other periodontopathogens, induce a host immune response, which in turn leads to a destructive inflammatory process [6,7].

Over the past two decades, there has been increasing interest in the potential human health benefits of natural compounds [8]. Polyphenols, which are well known for their antioxidant properties, contribute to the protection of deoxyribonucleic acid (DNA) and macromolecules (lipids and proteins) and can prevent some types of cancers, cardiovascular diseases, and other disorders associated with oxidative stress [9,10]. These natural compounds are members of a large class of organic molecules that are widely distributed in the plant kingdom and, as such, are an integral part of the daily diet of humans [11,12]. Since polyphenols have been reported to possess antimicrobial and anti-inflammatory properties, they may be of interest as therapeutic agents for controlling periodontal diseases, which involve both pathogenic bacteria and host immune responses.

**Figure 1:**
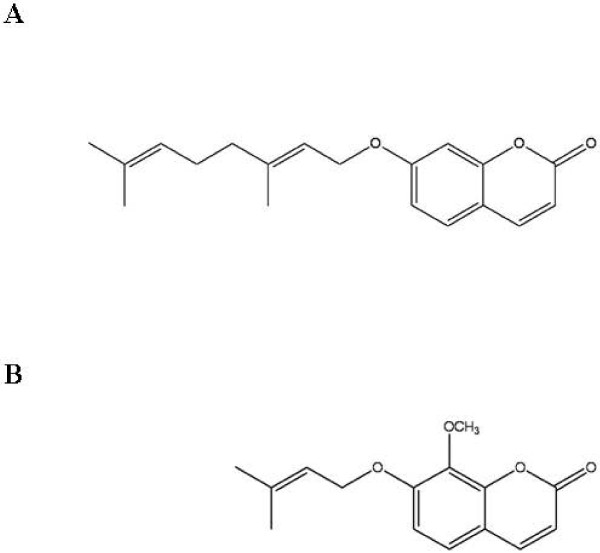
Chemical structures of auraptene (A) and lacinartin (B).

Auraptene and lacinartin are polyphenols that belong to the coumarin family [13,14]. While the chemical structures of these two compounds are similar, lacinartin has a methoxy group on the benzene ring and an isopentenyloxy side chain (Figure 1). Auraptene, which is also known as 7-geranyloxycoumarin, was first isolated in the 1930s by Komatsu et al. [15]. It is the most abundant naturally occurring prenyloxycoumarin and is mostly found in *Citrus* fruits [13,16,17]. Auraptene has been reported to possess antioxidant, anti-inflammatory, antibacterial, and anti-cancer properties [18,19] while little is known about lacinartin. 

To the best of our knowledge, no one has investigated the potential beneficial effects of auraptene and lacinartin on oral health. We hypothesized that auraptene and lacinartin may be promising natural compounds that could be used to prevent and treat periodontal diseases. We thus evaluated the effects of these compounds on the growth, biofilm formation/desorption, and adherence to human oral epithelial cells of *P. gingivalis*. We also investigated their anti-inflammatory properties using a macrophage model as well as their ability to inhibit MMP-9 and *P. gingivalis* collagenase.

## Materials and methods

### Compounds

Auraptene was purchased from Sigma-Aldrich Corp. (St. Louis, MO, USA). Lacinartin, an oxyisopentenylated coumarin, was produced using a previously reported procedure [20]. Briefly, commercially available propiolic acid and pyrogallol were condensed by concentrated H_2_SO_4_ catalysis into daphnetin via a Pechmann reaction. The daphnetin was then selectively alkylated on position 7 of the coumarin ring with 3,3-dimethylallyl bromide and 1,8-diazabicyclo[5.4.0]undec-7-ene (DBU). It was then methylated on position 8 with methyl iodide and triethylamine to yield lacinartin. The final yield was 62%. Stock solutions of auraptene and lacinartin were prepared in dimethyl sulfoxide (10 mg/ml) and stored at 4°C in the dark.

### Effect on *Porphyromonas gingivalis* growth

*P. gingivalis* ATCC 33277 was purchased from the American Type Culture Collection (Manassas, VA, USA). Bacteria were routinely grown in Todd-Hewitt broth (BBL Microbiology Systems, Mississauga, ON, Canada) supplemented with 20 μM hemin and 0.0001% vitamin K (THB-HK) at 37°C under anaerobic conditions (80% N_2_/10% H_2_/10% CO_2_) for 24 h. The effect of auraptene and lacinartin on *P. gingivalis* growth was assessed in two different culture media using a microplate dilution assay. THB-HK contained excess iron, while Mycoplasma broth base (MBB; BBL Microbiology Systems) supplemented with 10 μM hemin (MMB-H) contained limited iron. Briefly, 24-h cultures of *P. gingivalis* in THB-HK, or MBB-H were diluted in fresh broth medium to obtain an optical density of 0.2 at 660 nm (OD_660_). Equal volumes (100 μl) of *P. gingivalis* suspension and auraptene or lacinartin (0, 12.5, 25, 50, 100 μg/ml) in THB-HK, or MBB-H were mixed in the wells of 96-well plates (Sarstedt, Newton, NC, USA). Wells with no *P. gingivalis*, auraptene, or lacinartin were used as controls. After a 48-h incubation at 37°C under anaerobic conditions, bacterial growth was determined by measuring the OD_660_ using a microplate reader.

### Effect on *P. gingivalis* biofilm formation/desorption

*P. gingivalis* was grown in THB-HK supplemented or not with auraptene or lacinartin as described above. After a 48-h incubation under anaerobic conditions, spent medium and free-floating bacteria were removed by aspiration using a 26 G needle, and the wells were washed three times with 50 mM phosphate-buffered saline (PBS) pH 7.0. The biofilms were stained with 100 μl of 0.02% crystal violet for 15 min. The wells were then washed three times with PBS to remove unbound dye and were dried for 2 h at 37°C. Ethanol (100 μl, 95% (v/v)) was added to the wells, and the plate was shaken for 10 min to release the dye from the biofilms. The absorbance at 550 nm (A_550_) was measured to quantify biofilm formation. We also investigated the capacity of auraptene and lacinartin to promote the desorption of a *P. gingivalis* biofilm. Briefly, a 48-h *P. gingivalis* biofilm was prepared as described above and was treated for 2 h with auraptene or lacinartin at final concentrations ranging from 0 to 100 μg/ml. The biofilms were stained with crystal violet as described above. All the above assays were performed in triplicate.

### Effect on *P. gingivalis* adherence to oral epithelial cells

*P. gingivalis* cells were first labeled with fluorescein isothyocyanate (FITC). Briefly, a 10-ml aliquot of a 24-h culture (THB-HK) of *P. gingivalis* was centrifuged at 7000 x *g* for 10 min, and the pellet was suspended in 12 ml of 0.5 M NaHCO_3_ (pH 8) containing 0.03 mg/ml FITC. The bacterial suspension was incubated in the dark at 37°C for 30 min with constant shaking. The bacteria were then washed three times by centrifugation (7000 x *g* for 5 min) and were suspended in the original volume of PBS. The immortalized human oral epithelial cell line GMSM-K was kindly provided by Dr. Valerie Murrah (University of North Carolina, Chapel Hill, NC, USA). The epithelial cells were cultured in Dulbecco’s modified Eagle’s medium (DMEM) supplemented with 4 mM L-glutamine (HyClone Laboratories, Logan, UT, USA), 10% heat-inactivated fetal bovine serum (FBS; Sigma Aldrich Corp.), and 100 μg/ml of penicillin G/streptomycin at 37°C in a 5% CO_2_ atmosphere until they reached confluence. The cells were harvested by gentle trypsinization with 0.05% trypsin-ethylenediaminetetraacetic acid (Invitrogen, Grand Island, NY, USA) at 37°C and were suspended in DMEM (without FBS). Aliquots of cell suspension (100 μl, 1.5 x 10^6^ cells/ml) were placed in the wells of 96-well black plates (Greiner Bio-One, St. Louis, MO, USA). After an overnight incubation to allow the formation a confluent monolayer, spent medium was aspirated, 100 μl of formaldehyde (3.7%) was added to the wells, and the plate was incubated at room temperature for 15 min. The formaldehyde was removed by aspiration and the wells were washed three times with PBS. Filtered 1% BSA (100 μl) was added to each well, and the plate was incubated for 30 min at 37°C in a 5% CO_2_ atmosphere. The wells were washed once with PBS, 100 μl of auraptene or lacinartin was added to each cell (final concentrations ranging from 0 to 100 μg/ml), and the plates were incubated for 30 min. The auraptene and lacinartin were not cytotoxic at these concentrations (data not shown). The FITC-labeled *P. gingivalis* cells were then added (100 μl) to the wells, and the plates were incubated in the dark for a further 90 min at 37°C under anaerobic conditions. Unbound bacteria were removed by aspiration, and the wells were washed three times with PBS. Relative fluorescence units (RUF; excitation wavelength 495 nm; emission wavelength 525 nm) corresponding to the degree of bacterial adherence were determined using a microplate reader. Control wells without auraptene or lacinartin were used to determine 100% adherence values. Wells containing only cells and auraptene or lacinartin were also prepared to determine the autofluorescence values of the two compounds. The assays were run in triplicate.

### Anti-inflammatory properties in a macrophage model

U937 human monocytes (ATCC CRL-1593.2), a monoblastic leukemia cell line, were purchased from the American Type Culture Collection (Manassas, VA, USA). The cells were cultivated at 37°C in a 5% CO_2_ atmosphere in Roswell Park Memorial Institute 1640 medium (RPMI-1640; HyClone Laboratories) supplemented with 10% heat-inactivated FBS and 100 μg/ml of penicillin G/streptomycin. The monocytes (2.5 x 10^5^ cells/ml) were then incubated in RPMI-FBS (1%) containing 10 ng/ml of phorbol myristic acid (PMA; Sigma Aldrich Corp.) for 48 h to induce differentiation into adherent macrophage-like cells. Following the PMA treatment, the medium was replaced with fresh medium, and the differentiated cells were incubated for an additional 24 h prior to use. The macrophages were incubated with auraptene or lacinartin (6.25 to 50 μg/ml) at 37°C in a 5% CO_2_ atmosphere for 2 h. They were then stimulated with 1 μg/ml of *Aggregatibacter actinomycetemcomitans* ATCC 29522 (serotype b) lipopolysaccharide (LPS) isolated using the procedure described by Darveau and Hancock [21]. After a 24-h incubation at 37°C in a 5% CO_2_ atmosphere, the culture medium supernatants were collected and were stored at –20°C until used. Cells incubated in culture medium with or without auraptene or lacinartin but not stimulated with LPS were used as controls. Commercial enzyme-linked immunosorbent assay (ELISA) kits (R&D Systems, Minneapolis, MN, USA) were used to quantify IL-8, TNF-α, MMP-8, and MMP-9 concentrations in the cell-free culture supernatants according to the manufacturer’s protocols. The absorbance at 450 nm was read using a microplate reader with the wavelength correction set at 550 nm.

### Inhibition of MMP-9 and *P. gingivalis* collagenase activity

Human recombinant MMP-9 (active form) purchased from Calbiochem (San Diego, CA, USA) was diluted in reaction buffer (50 mM Tris-HCl, 150 mM NaCl, 5 mM CaCl_2_, and 0.02% Brij 35) to a concentration of 1 μg/ml and was incubated for 18 h in the absence or presence of auraptene or lacinartin (0-100 μg/ml) and fluorogenic substrate (100 μg/ml). To determine the effect of auraptene and lacinartin on *P. gingivalis* collagenase activity, a 48-h THB-HK culture was centrifuged at 10 000 x *g* for 10 min. The supernatant was then incubated for 18 h in the absence or presence of auraptene or lacinartin (0-100 μg/ml) and fluorogenic substrate (100 μg/ml). Gelatin DQ^TM^ and collagen DQ^TM^ (Molecular Probes, Eugene, OR, USA) were used to quantify MMP-9 and *P. gingivalis* collagenase activities, respectively. The assay mixtures were incubated for 18 h at 37°C for MMP-9 and at room temperature for *P. gingivalis* collagenase. The fluorescence was measured after 4 h using a microplate reader with the excitation and emission wavelengths set at 495 nm and 525 nm, respectively. Fluorescent substrates alone or with auraptene and lacinartin were used as controls. Specific inhibitors of MMP-9 (0.025 μM GM6001) and *P. gingivalis* collagenase (1 μM leupeptin) were tested. The assays were run in triplicate.

### Statistical analysis

Results are expressed as the means ± standard deviations of three independent experiments. The data were analyzed using the Student’s t-test. A *p* value ≤ 0.05 was considered statistically significant.

## Results

**Figure 2:**
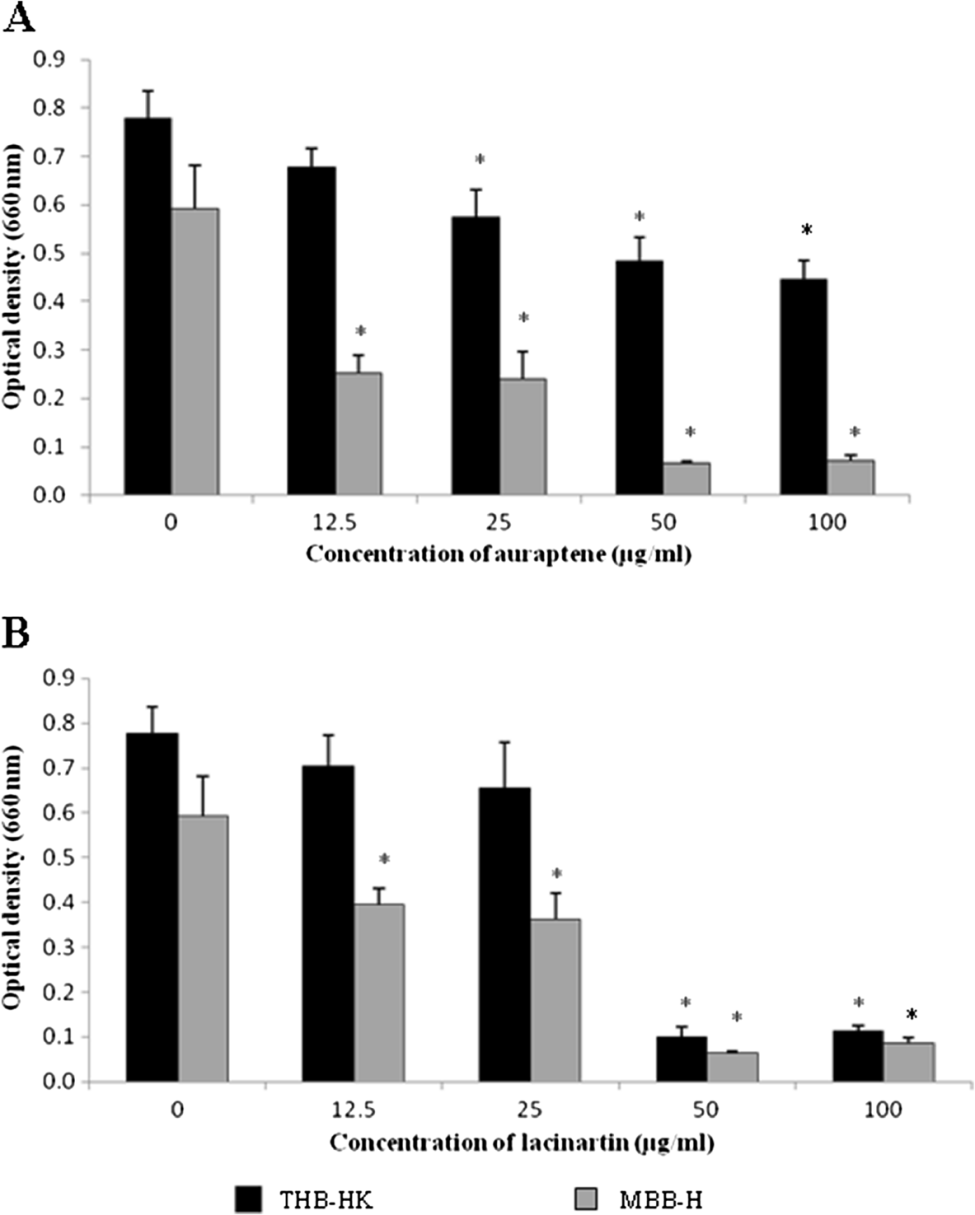
** Effect of auraptene (A) and lacinartin (B) on the growth of *P. gingivalis* in a complex medium (THB-HK) and under iron-limiting conditions in MBB-H.** Values are expressed as means ± standard deviations of triplicate assays for a minimum of three independent experiments. Data were analyzed using the Student’s *t*-test (*: *p* ≤ 0.05 *vs.* control without auraptene or lacinartin).

Lacinartin (50 and 100 μg/ml) almost completely inhibited the growth of *P. gingivalis* in THB-HK (Figure. 2B), while the highest concentration of auraptene tested (100 μg/ml) only reduced growth by 42% (Figure. 2A). Bacterial growth was slightly lower in MBB-H (OD_660_ = 0.59), which represents an iron poor condition for *P. gingivalis*, than in THB-HK (OD_660_ = 0.78) (Figure. 2). The lowest concentration of auraptene tested (12.5 μg/ml) inhibited growth by 58% in MBB-H, while such a concentration had no significant inhibitory effect in THB-HK (Figure. 2A). Almost complete inhibition was observed at higher concentrations (50 and 100 μg/ml). As for auraptene, lacinartin seemed to be more effective for inhibiting growth of *P. gingivalis* in iron-limiting conditions.

**Figure 3:**
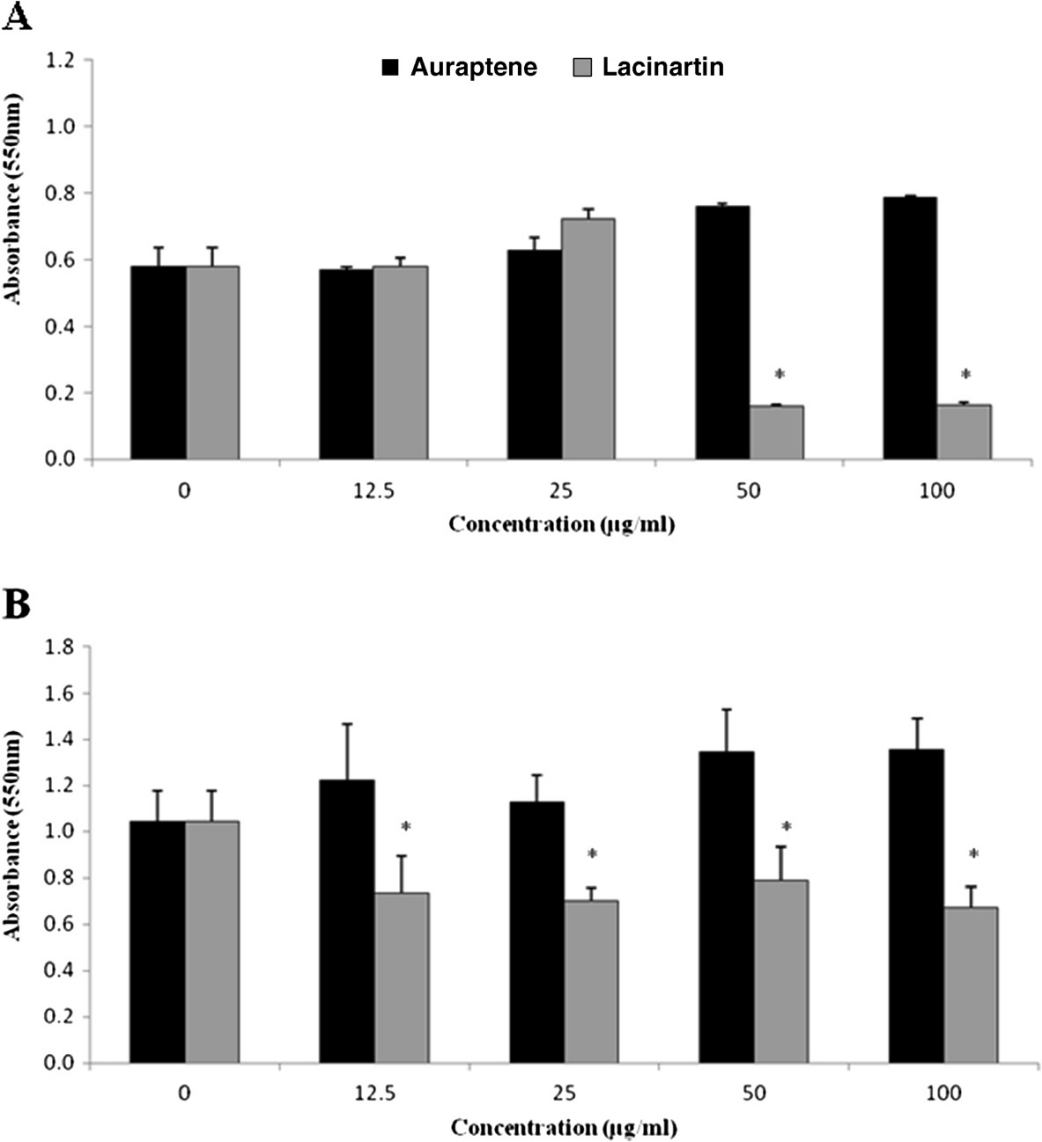
** Effect of auraptene and lacinartin on *P. gingivalis * biofilm formation (A) and on desorption of a pre-formed *P. gingivalis * biofilm (B).** Values are expressed as means ± standard deviations of triplicate assays for a minimum of three independent experiments. Data were analyzed with the Student’s *t*-test (*: *p* ≤ 0.05 *vs.* control without auraptene or lacinartin).

Auraptene had no obvious inhibitory effect on *P. gingivalis* biofilm formation or desorption (Figure. 3). On the contrary, high concentrations (50 and 100 μg/ml) of auraptene appear to increase biofilm formation (Figure. 3A). Lacinartin at 50 and 100 μg/ml inhibited biofilm formation by *P. gingivalis* by approximately 75% (Figure. 3A). In addition, lacinartin (12.5-100 μg/ml) caused approximately one-third of the biofilm to desorb (Figure. 3B).

**Figure 4:**
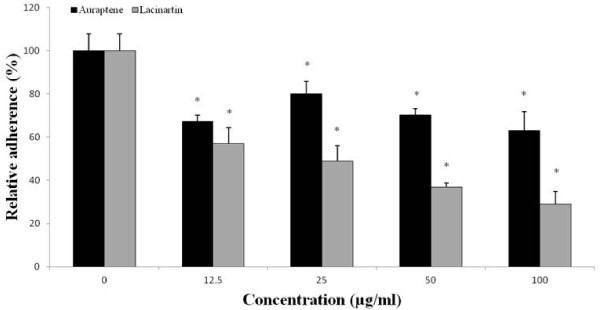
** Effect of auraptene and lacinartin on the adherence of *P. gingivalis * to human oral epithelial cells.** Values are expressed as means ± standard deviations of triplicate assays for a minimum of three independent experiments. A value of 100% was assigned to the control (no auraptene; no lacinartin). Data were analyzed with the Student’s *t*-test (*: *p* ≤ 0.05 *vs.* control without auraptene or lacinartin).

Auraptene and lacinartin both dose-dependently inhibited bacterial adhesion to oral epithelial cells (Figure. 4). At the lowest concentration tested (12.5 μg/ml), auraptene and lacinartin reduced the adherence of *P. gingivalis* to epithelial cells by 33% and 43%, respectively, while 100 μg/ml of auraptene and lacinartin reduced adherence by 37% and 71%, respectively (Figure. 4).

**Figure 5:**
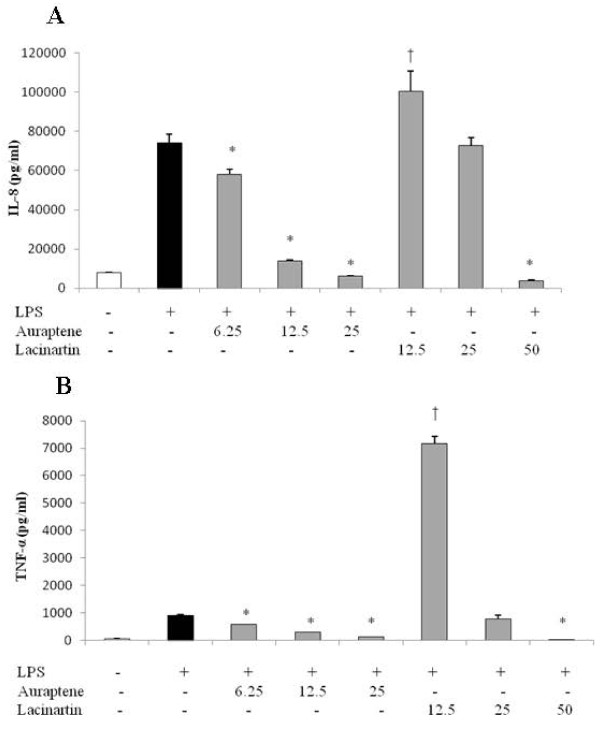
** Effect of auraptene and lacinartin on the secretion of IL-8 (A) and TNF-α (B) by human macrophages stimulated with *A. actinomycetemcomitans * LPS (1 μg/ml).** Values are expressed as means ± standard deviations of triplicate assays for a minimum of three independent experiments. Data were analyzed with the Student’s *t*-test (*: *p* ≤ 0.05 *vs.* untreated control, ^†^: *p* ≤ 0.05 vs. control without auraptene or lacinartin).

**Figure 6:**
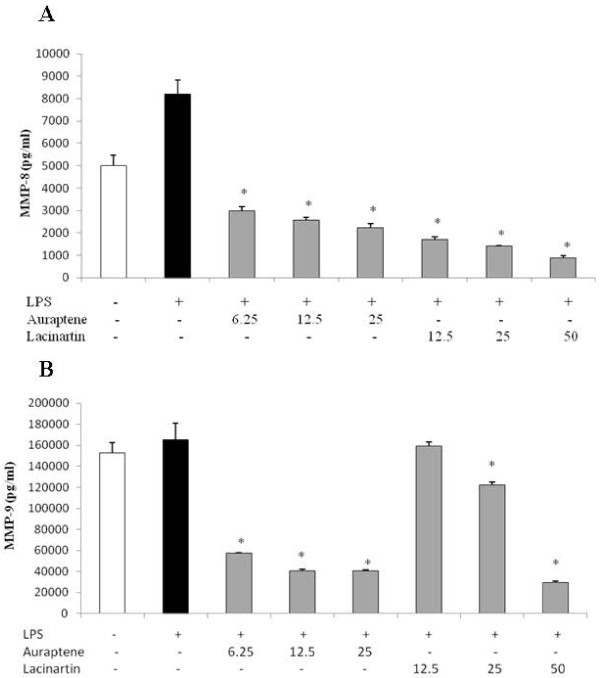
** Effect of auraptene and lacinartin on the secretion of MMP-8 (A) and MMP-9 (B) by human macrophages stimulated with *A. actinomycetemcomitans * LPS (1 μg/ml).** Values are expressed as means ± standard deviations of triplicate assays for a minimum of three independent experiments. Data were analyzed with the Student’s *t*-test (*: *p* ≤ 0.05 *vs.* untreated control).

The highest non-cytotoxic concentrations of auraptene and lacinartin that can be used to evaluate their effect on the inflammatory response of a human macrophage model stimulated with LPS were 25 and 50 μg/ml, respectively (data not shown). Following a 2-h pretreatment of the model with auraptene (6.25, 12.5, and 25 μg/ml) or lacinartin (12.5, 25, and 50 μg/ml), macrophages were stimulated with LPS to induce an inflammatory response (cytokine and MMP secretion). Auraptene and lacinartin both had a significant inhibitory effect on IL-8 and TNF-α secretion. At the lowest concentration tested (6.25 μg/ml), auraptene reduced IL-8 and TNF-α secretion by 22% and 37%, respectively, compared to untreated cells, while at the highest concentration tested (25 μg/ml), it inhibited IL-8 and TNF-α secretion by 92% and 85%, respectively (Figure. 5). As shown in Fig. 5, the highest concentration of lacinartin tested (50 μg/ml) reduced IL-8 and TNF-α secretion by 95% and 99%, respectively, while 12.5 and 25 μg/ml of lacinartin increased the secretion of both cytokines, likely due to a synergistic effect of LPS and lacinartin, since lacinartin alone had no effect (data not shown). Auraptene and lacinartin both reduced MMP-8 and MMP-9 secretion, sometimes below basal levels (Figure. 6). Auraptene reduced MMP-8 secretion by 63% (6.25 μg/ml), 69% (12.5 μg/ml), and 73% (25 μg/ml), while lacinartin reduced MMP-8 secretion by 79% (12.5 μg/ml), 83% (25 μg/ml), and 89% (50 μg/ml) (Figure. 6A). At their highest concentrations tested, auraptene (25 μg/ml) reduced MMP-9 secretion by 76% (25 μg/ml) (Figure. 6B), while lacinartin (50 μg/ml) reduced MMP-9 secretion by 82% (Figure. 6B).

After demonstrating that auraptene and lacinartin can decrease MMP secretion in a macrophage model, we evaluated their effect on proteinase activity. Both auraptene and lacinartin (12.5 μg/ml) reduced MMP-9 activity by 74% (Table 1). Auraptene had no inhibitory effect on *P. gingivalis* collagenase activity while 12.5 μg/ml and 100 μg/ml of lacinartin reduced collagenase activity by 20% and 64%, respectively (Table 1).

**Table 1:** **Effect of auraptene and lacinartin on the activity of MMP-9 and *P. gingivalis* collagenase**

**Compound**	**MMP-9 (% activity)**	**Collagenase (% activity)**
None	100 ± 1	100 ± 3
Commercial inhibitor^1^	56 ± 1	6 ± 5
Auraptene 100 μg/ml	27 ± 1	110 ± 4
50	27 ± 1	113 ± 5
25	27 ± 3	113 ± 1
12.5	26 ± 1	107 ± 10
Lacinartin 100 μg/ml	19 ± 6	36 ± 3
50	25 ± 1	49 ± 1
25	31 ± 2	69 ± 1
12.5	26 ± 3	80 ±1

^1^Inhibitor of MMP-9: GM6001 (0.025 μM); inhibitor of *P. gingivalis* collagenase: leupeptin (1 μM).

## Discussion

Periodontal diseases are polymicrobial infections and are the most common chronic inflammatory disorders in adults [22]. Periodontitis is induced by a specific group of Gram-negative anaerobic bacteria and is the major cause of tooth loss in adults [23]. Over the past two decades, natural compounds with antibacterial and anti-inflammatory properties have received considerable attention as new therapeutic agents for the treatment of periodontal infections. In this study, we investigated the potential of auraptene and lacinartin for preventing and treating periodontal diseases.

We first showed that lacinartin and to a lesser extent auraptene reduced *P. gingivalis* growth. This is the first report indicating that lacinartin possesses anti-bacterial properties. Previous studies have shown that auraptene has antibacterial properties against *Helicobacter pylori*[24,25]*.* The exact mechanism by which lacinartin and auraptene inhibit bacterial growth is unknown. However, other natural coumarins (novobiocin and clorobiocin) inhibit deoxyribonuclease gyrase activity, which results in bacteria death [14,26]. In addition, we showed that auraptene and lacinartin inhibit growth more effectively under iron-limiting conditions, requiring much lower concentrations to significantly reduce the growth of *P. gingivalis*. Our results are in agreement with those of Mladnka et al. [27], who showed that coumarins possess iron-chelating properties. Additional studies are required to investigate interactions between iron and auraptene and lacinartin.

We also showed that lacinartin, but not auraptene, inhibits biofilm formation by *P. gingivalis*. Lacinartin also caused the desorption of a pre-formed *P. gingivalis* biofilm. To the best of our knowledge, this is the first report regarding the inhibitory effects of lacinartin on bacterial biofilms. Auraptene and lacinartin prevented the adherence of *P. gingivalis* to oral epithelial cells to a significant degree. Epithelial cells act as a physical barrier, and bacterial adherence to these host cells may be a critical step for the initiation of periodontal diseases [28]. Given its ability to reduce growth of *P. gingivalis* and its adherence to epithelial cells, lacinartin may be a promising therapeutic candidate through its action on different targets.

Polyphenols reduce inflammatory mediator secretion and, as such, inflammation-mediated damage [29]. We showed that auraptene markedly reduces IL-8 and TNF-α secretion by LPS-stimulated macrophages. Our results are in agreement with those of Genovese et al., who reported that auraptene inhibits the release of TNF-α by RAW 264.7 macrophages [30]. To our knowledge, no one has investigated the anti-inflammatory properties of lacinartin. We showed that 12.5 μg/ml of lacinartin induced IL-8 and TNF-α secretion, likely due to a synergistic interaction between LPS and lacinartin. On the other hand, 50 μg/ml of lacinartin significantly inhibited IL-8 and TNF-α secretion. We also showed that auraptene and lacinartin reduced MMP-8 and MMP-9 secretion. These results are in agreement with those of a study by Epifano et al., who reported that auraptene inhibits MMP-7 secretion by HT-29 epithelial cells [18]. Since MMP release and cytokine secretion are associated with tooth-supporting tissue destruction, our results suggested that both compounds may contribute to reducing host cell damage, including bone resorption [5,31]. The mechanisms by which auraptene and lacinartin reduce inflammatory mediator secretion are unknown, but previous studies have shown that coumarins can block the activation of nuclear factor-κB and inhibit kinase pathways (Akt/PKB) [26]. Considering that gingival fibroblasts may also play a significant role in periodontal tissue destruction through cytokine-inducible MMP secretion, future studies should investigate the effects of auraptene and lacinartin on this cell type.

We further showed that auraptene and lacinartin reduce MMP-9 activity while only lacinartin inhibits *P. gingivalis* collagenase activity. Auraptene has previously been shown to inhibit MMP-7 activity [32]. These observations suggest that these coumarins may contribute to reducing tissue destruction.

## Conclusions

In conclusion, our study provided new information on auraptene and lacinartin indicating that they possess an array of interesting antimicrobial, anti-adhesion, anti-inflammatory and anti-protease properties that may be useful for the prevention and treatment of periodontal diseases. Since auraptene and lacinartin act on both etiologic factors of periodontal diseases (periodontopathogens and the host inflammatory response), they may be an alternative to traditional antimicrobials. Further studies are required to investigate the mechanisms of these coumarins, especially the mechanisms involved in their anti-inflammatory activity.

## Competing interests

The authors declare that they have no competing interests.

## Authors’ contributions

All authors contributed equally in data acquisition and in writing of the manuscript. All the authors read and approved the final version of the manuscript.

## Pre-publication history

The pre-publication history for this paper can be accessed here:

http://www.biomedcentral.com/1472-6882/12/80/prepub
